# Comparative volatiles profiling of two marjoram products via GC-MS analysis in relation to the antioxidant and antibacterial effects

**DOI:** 10.1038/s41598-024-78674-y

**Published:** 2024-11-13

**Authors:** Mostafa B. Abouelela, Enas M. Shawky, Omayma Elgendy, Mohamed A. Farag, Mostafa H. Baky

**Affiliations:** 1 Pharmacognosy Department, Faculty of Pharmacy, Egyptian Russian University, Badr, Cairo 11829 Egypt; 2 Pharmacognosy Department, College of Pharmacy, Cairo University, Cairo, 11562 Egypt; 3 Pharmacognosy Department, College of Pharmacy, Egyptian Russian University, Badr, Cairo 11829 Egypt

**Keywords:** Antibacterial, DPPH, GC-MS, HS-SPME, *Origanum majorana*, Sweet oregano, Total phenolic, Total flavonoid, Metabolomics, Mass spectrometry

## Abstract

Marjoram (*Origanum majorana* L.), also known as “sweet marjoram” or “sweet oregano” is a Mediterranean herbaceous perennial herb cultivated in Egypt and widely consumed as an herbal supplement for treatment of several ailments. The main goal of this study was to assess volatiles’ variation in marjoram samples collected from two different widely consumed commercial products using two different extraction techniques viz. head space solid phase microextraction (HS-SPME) and petroleum ether using gas chromatography mass spectrometry (GC-MS) analysis and multivariate data analysis. A total of 20 major aroma compounds were identified in samples extracted with HS-SPME found enriched in monoterpene hydrocarbons and oxygenated compounds. The major volatiles included *β*-phellandrene (20.1 and 14.2%), *γ*-terpinene (13.4 and 11.7%), 2-bornene (12.3 and 11.5%), *p*-cymene (9.8 and 4.6%) terpenen-4-ol (16.4 and 7.5%), sabinene hydrate (16.02 and 8.8%) and terpineol (4.2 and 3.2%) in MR and MI, respectively. Compared with HS-SPME, 51 aroma compounds were identified in marjoram samples extracted with petroleum ether, found more enriched in aliphatic hydrocarbons (42.8 and 73.8%) in MR and MI, respectively. While a higher identification score was observed in the case of solvent extraction, SPME appeared to be more selective in the recovery of oxygenated terpenes to account more for marjoram aroma. Multivariate data analysis using principal component analysis (PCA) revealed distinct discrimination between volatile composition of both marjoram samples. The total phenolic and flavonoid contents in marjoram samples were at (111.9, 109.1 µg GA/mg) and (18.3, 19.5 µg rutin eq/mg) in MR and MI, respectively. Stronger antioxidant effects were observed in MR and MI samples with IC_50_ at 45.5 and 56.8 µg/mL respectively compared to IC_50_ 6.57 µg/mL for Trolox as assayed using DPPH assay. Moderate anti-bacterial effect was observed in MR and MI samples and expressed as a zone of inhibition mostly against *Bacillus subtilis* (16.03 and 15.9 mm), *B. cereus* (12.9 and 13.7 mm), *Enterococcus faecalis* (14.03 and 13.97 mm), and *Enterobacter cloacae* (11.6 and 11.6 mm) respectively.

## Introduction

Natural products are considered as an important spirit for the discovery of novel pharmaceutical active products^1^. Medicinal plants play a chief role in various disciplines, ranging from the food industry to the fragrance and cosmetics sector, as well as in different medicinal and pharmaceutical applications^2–4^. Labiatae is one of the major herbal families that encompasses ca. 224 genera and 5600 species of well-known aromatic plants with potential biological and health benefits^5^. The genus *Origanum* is one of the most important genera belonging to the family Labiatae that includes 42 aromatic species and 18 hybrids widely distributed throughout Europe, Asia, and North Africa^6^. Marjoram (*Origanum majorana* L.), also known as “sweet marjoram” or “sweet oregano” is a Mediterranean herbaceous perennial herb widely cultivated in Egypt^7^, Europe, northern America, Greece, France, and Asia^8^. Marjoram is well known for its economic importance being included in pharmaceutical, cosmetic, food production^9^, and food preservation^10^. Moreover, marjoram is used as a condiment and spice for special flavoring in foods such as meat, fish, soups, sauces, and canned foods^11^.

Marjoram is traditionally used to treat several ailments. Marjoram leaves are widely used as anti-anxiety, anticonvulsant, and anti-gout^12^. Leaf decoction is used in the treatment of respiratory infections and as an anti-diabetic^13^, whereas leaf infusion is reported for the management of hypertension^14^. Mixed leaf and flower infusion exhibited a calming, antispasmodic effect, and relief colds, fever, and headaches^15^, whereas, blended leaf and stem are known to be effective against rheumatism, stomach pain, headache, cough, insomnia, and as antipyretic^16^, meanwhile, the whole plant has a sedative effect^17^. Marjoram leaf infusion and decoction are used as sedatives and to relieve nerve pain in Italian traditional medicine^18^. Moreover, marjoram was used for the treatment of digestive disorders, and bug bites, and as a disinfectant^19^.

Considering its favored and rich aroma, marjoram has been previously investigated for its essential oil and phenolics composition^13^. There are various essential oils in two different commercial products using two different extracts such as linalyl acetate and santalene in HS-SPME and *γ*-sitosterol and longipinane in petroleum ether extract. In addition to essential oils, several phenolics were identified from marjoram including luteolin-7-*O*-*β*-glucuronide and methyl rosmarinate which were isolated from the aqueous acetone extracts of the marjoram dried herb cultivated in Poland^20^.

Recently, the growth of consumer demand for herbal products with exact composition revealed warrants for the development of analytical tools to assess and assure their quality^21^. Metabolomics tools are widely used for the quality assessment of herbal products owing to their large scope of metabolites detection^22^. Among metabolomics techniques, GC-MS analysis is well adopted for the profiling of volatiles^23^. Several extraction techniques are widely used for essential oil recovery from herbal materials among which headspace solid phase microextraction (HS-SPME) and solvent extraction are widely used^24^. Solid phase microextraction (SPME) technique has been successfully used for more than three decades in aroma profiling. It is based upon the potential use of a wide variety of polymeric organic fibers which can be either synthesized or be purchased from commercially available companies^25–27^. To aid in comparison among different samples of origin or derived using various extraction methods, multivariate data analysis including principal component analysis (PCA) is widely used to better assess such data matrix^21^.

The main goal of this study was to assess volatiles’ differences from marjoram samples collected from two different widely consumed commercial products in the Egyptian market (MR and MI) using two different extraction techniques viz. HS-SPME and solvent extraction. Moreover, assessment of total phenolics and flavonoids in marjoram methanol extract was performed to account for the non-volatile phenolic portion in marjoram. In vitro, assays of antioxidant and antibacterial potential in marjoram samples were performed.

## Results and discussion

### Volatile chemical composition of marjoram samples extracted using HS-SPME via GC-MS analysis

**Fig. 1:**
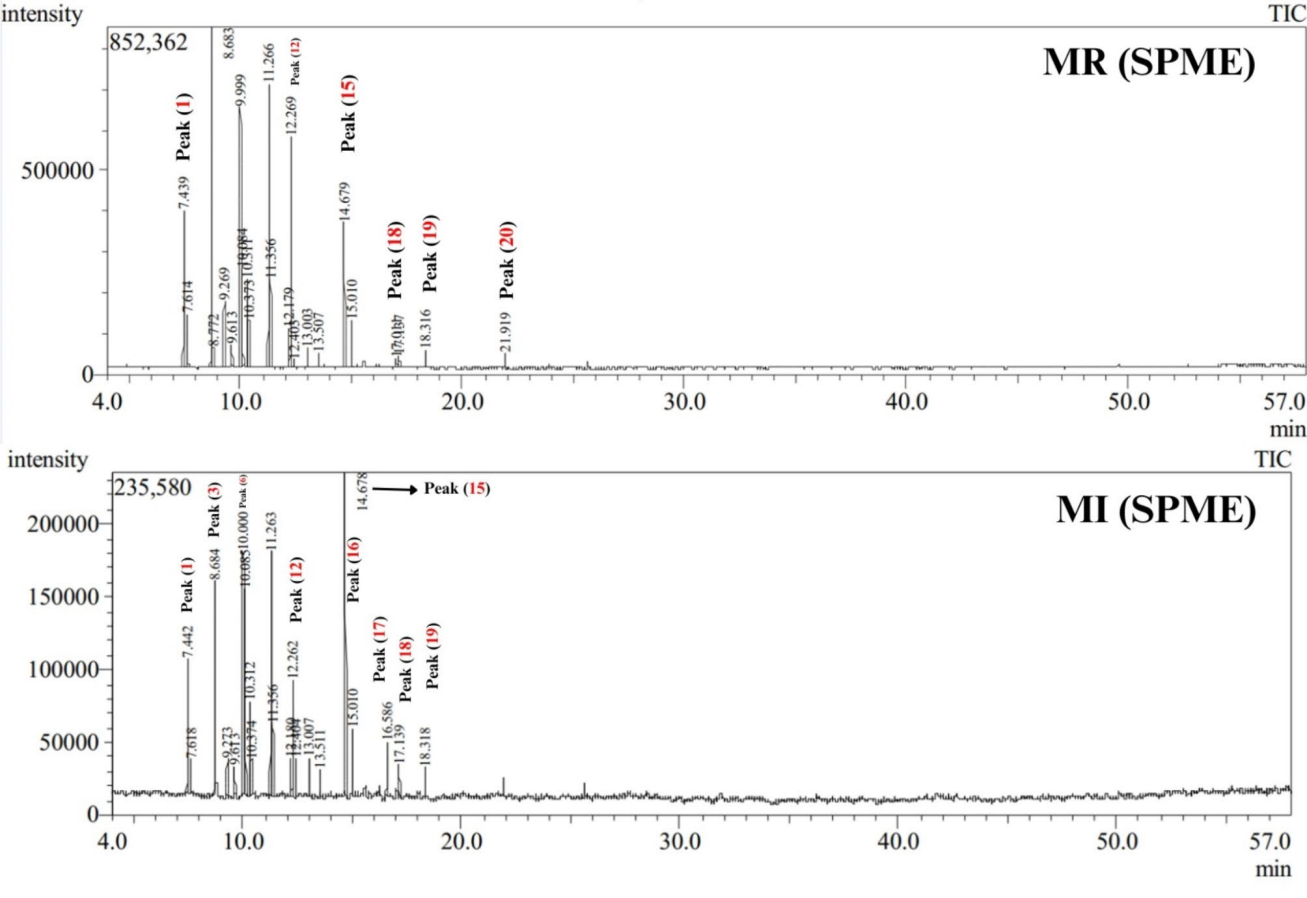
Representative GC chromatogram of volatiles identified in marjoram commercial products (MR and MI) analyzed after extraction with HS-SPME and peak numbers as listed in Table 1, peak (1): *α*-Thujene, peak (3): *β*-Phellandrene, peak (6): 2-Bornene, peak (12): *cis*-Sabinene hydrate, peak (15): Terpinen-4-ol, peak (16): Terpinyl formate, peak (17):, peak (18): (+)-3-Carene, peak (19): 4-Terpinenyl acetate, peak (20): santalene.

The volatile profiles in two widely consumed commercial marjoram products were analyzed using HS-SPME coupled to GC-MS analysis. A total of 20 peaks (Fig. 1) were identified belonging to several chemical classes’ including alcohol, ester, ketone, monoterpene hydrocarbon, oxygenated monoterpene, and sesquiterpene hydrocarbon (Table 1).

**Table 1:** Relative area percentage (%) of volatile metabolites in two commercial marjoram leaf products analyzed via HS-SPME GC-MS (n = 3).

No	Rt	KI	Compound name	Class	R (%)	Sd	I (%)	sd
14	13.003	1041	4-Thujanol	Alcohol	1.66	0.04	2.93	0.01
15	14.678	1137	Terpinen-4-ol	Alcohol	7.58	0.03	16.47	0.03
Total alcohol					9.24	0.07	19.40	0.04
13	12.403	1272	Linalyl acetate	Ester	0.41	0.01	1.95	0.01
16	15.01	1330	Terpinyl formate	Ester	2.33	0.02	3.36	0.01
19	18.316	1327	4-Terpinenyl acetate	Ester	1.43	0.01	1.25	0.02
Total ester					4.17	0.04	6.56	.03
17	16.586	1158	Carvenone	Ketone	0.00	0.00	2.86	0.14
Total ketone					0.00	0.00	2.86	0.14
1	7.442	902	*α*-Thujene	Monoterpene hydrocarbon	6.91	0.02	6.17	0.02
2	7.618	948	*α*-Pinene	Monoterpene hydrocarbon	3.34	0.02	1.31	0.02
3	8.684	964	*β*-Phellandrene	Monoterpene hydrocarbon	20.10	0.05	14.26	0.03
4	9.273	943	*β*-Pinene	Monoterpene hydrocarbon	2.96	0.02	1.59	0.02
5	9.613	873	*β*-Thujene	Monoterpene hydrocarbon	0.92	0.02	1.19	0.02
6	10	932	2-Bornene	Monoterpene hydrocarbon	12.34	0.05	11.59	0.03
7	10.085	1042	*p*-Cymene	Monoterpene hydrocarbon	4.63	0.02	9.86	0.03
8	10.374	943	Camphene	Monoterpene hydrocarbon	2.32	0.01	1.62	0.03
9	11.266	998	*γ*-Terpinene	Monoterpene hydrocarbon	13.40	0.30	11.74	0.03
11	12.18	919	( +)-4-Carene	Monoterpene hydrocarbon	2.06	0.01	1.78	0.01
18	17.139	948	( +)-3-Carene	Monoterpene hydrocarbon	0.60	0.01	1.28	0.02
Total monoterpene hydrocarbon					69.59	0.51	62.39	0.26
10	11.356	1191	*γ-*Terpineol	Oxygenated monoterpene	4.24	0.02	3.28	0.02
12	12.262	1041	*cis*-Sabinene hydrate	Oxygenated monoterpene	11.78	0.02	5.59	0.02
Total oxygenated monoterpene					16.02	0.03	8.87	0.04
20	21.919	1211	Santalene	Sesquiterpene hydrocarbon	0.88	0.01	0.00	0.00
Total sesquiterpene hydrocarbon					0.88	0.01	0.00	0.00

Significance value bold.

#### Mono- and sesquiterpene hydrocarbons

Monoterpene hydrocarbons represented by 11 peaks were detected as the most abundant class in marjoram samples from the two commercial sources at levels of 69.5 and 62.4% in MR and MI samples. β-Phellandrene (peak 3) was identified as the major volatile compound in both samples at levels 20.1 and 14.2%, respectively. In contrast, p-cymene (peak 7) was detected at a higher level in the marjoram sample (MI) at 9.8% versus 4.6% in marjoram sample (MR). Moreover, comparable levels were detected in the case of *γ*-terpinene (peak 9) and 2-bornene (peak 6) in both samples MR and MI 12–13% and 11–12% and suggestive of no pattern in the monoterpene profile for each percentage. Unlike monoterpene hydrocarbons, sesquiterpenes were detected at trace levels, only in MR samples, and absent in MI samples.

Previous investigation of marjoram samples revealed the detection of α-thujene, α-terpineol, borneol, carvacrol, *β*-caryophyllene, eucalyptol, linalool, myrcene, *p*-cymene, phellandrene, sabinene, terpinene, and terpinolene^13^.

#### Alcohol, ketone, ester, and oxygenated monoterpenes

Alcohols represented by two peaks were detected at higher levels in MI samples at 19.4% compared to 9.2% in MR samples. Terpenen-4-ol (peak 15) was detected as the major alcohol in both marjoram samples at a two-folds higher level in MI (16.4%) compared to the MR sample (7.5%). Ketones represented by carvenone (peak 17) was detected only in MI samples at 2.8%. Likewise, esters represented by 3 peaks were detected in MI samples at 6.5% compared with 4.2% in MR samples, with terpinyl formate (peak 16) as th major form in both marjoram samples. In contrast, oxygenated monoterpenes were detected at a higher level in MR samples at 16.02% compared to 8.8% in MI samples. Sabinene hydrate (peak 12) was previously detected at high levels in *O. majorana*^7^ and was found abundant in MR at 11.8% compared to 5.6% in the MI sample. The oil composition of marjoram cultivated in India was reported by Raina and Negi^28^, revealing the presence of terpinen-4-ol, sabinene hydrate, *p*-cymene, sabinene, and *α*-terpineol as the most abundant volatiles. *γ*-Terpineol (peak 10) was detected in both marjoram samples at 3–4%. Compared to other previously reported studies, the essential oil composition of three marjoram accessions cultivated in Egypt was investigated using hydro-distillation technique^7^, with the major volatiles to include sabinene hydrate (15.4–34.3%), α-terpinene (8.9–18%), 4-terpineol (15.2–35%), terpinolene (10.3–11.8%), and sabinene (7.4–8.4%)^7^.

Moreover, the oil composition of marjoram cultivated in India was likewise studied by Raina and Negi (2012)^28^ revealing the detection of terpinen-4-ol, sabinene hydrate, *p*-cymene, sabinene, and *α*-terpineol as the most abundant volatiles. Marjoram leaf collected from Egypt extracted by hydro-distillation and supercritical CO_2_ using GC-MS analysis revealed the abundance of terpinen-4-ol and sabinene^29^. Terpinen-4-ol was detected as the most abundant volatile detected in marjoram essential oil^30^. The abundance of cis-sabinene is a chief determinant of high-quality essential oils in *O. majorana* (sweet oregano)^7^. In another study, analysis of essential oil composition in sweat marjoram revealed the detection of terpinene-4-ol (20.9%), linalool (15.7%), linalyl-acetate (13.9%), limonene (13.4%), and *α-*terpineol (8.57%)^31^. Previous reports indicated that carvacrol was abundant in Turkish marjoram, whereas Iranian variety was richer in linalyl acetate. Marjoram varieties grown in Reunion Island, Greece, and Egypt were rich in terpinen-4-ol and sabinenes^19^. In another study, marjoram essential oil was reported to be rich in carvacrol, thymol, terpinen-4-ol, *trans*-caryophyllene, γ-terpinene, and *p*-cymene^32^. A study on the essential oil composition of the Iranian variety revealed the detection of linalool, thymol, *p*-cymene, terpinen-4-ol, sabinene, *β*-myrcene, *β-*caryophyllene, and *γ-*terpinene^33^.

### Volatile chemical composition of marjoram samples extracted using petroleum ether via GC-MS analysis

**Fig. 2:**
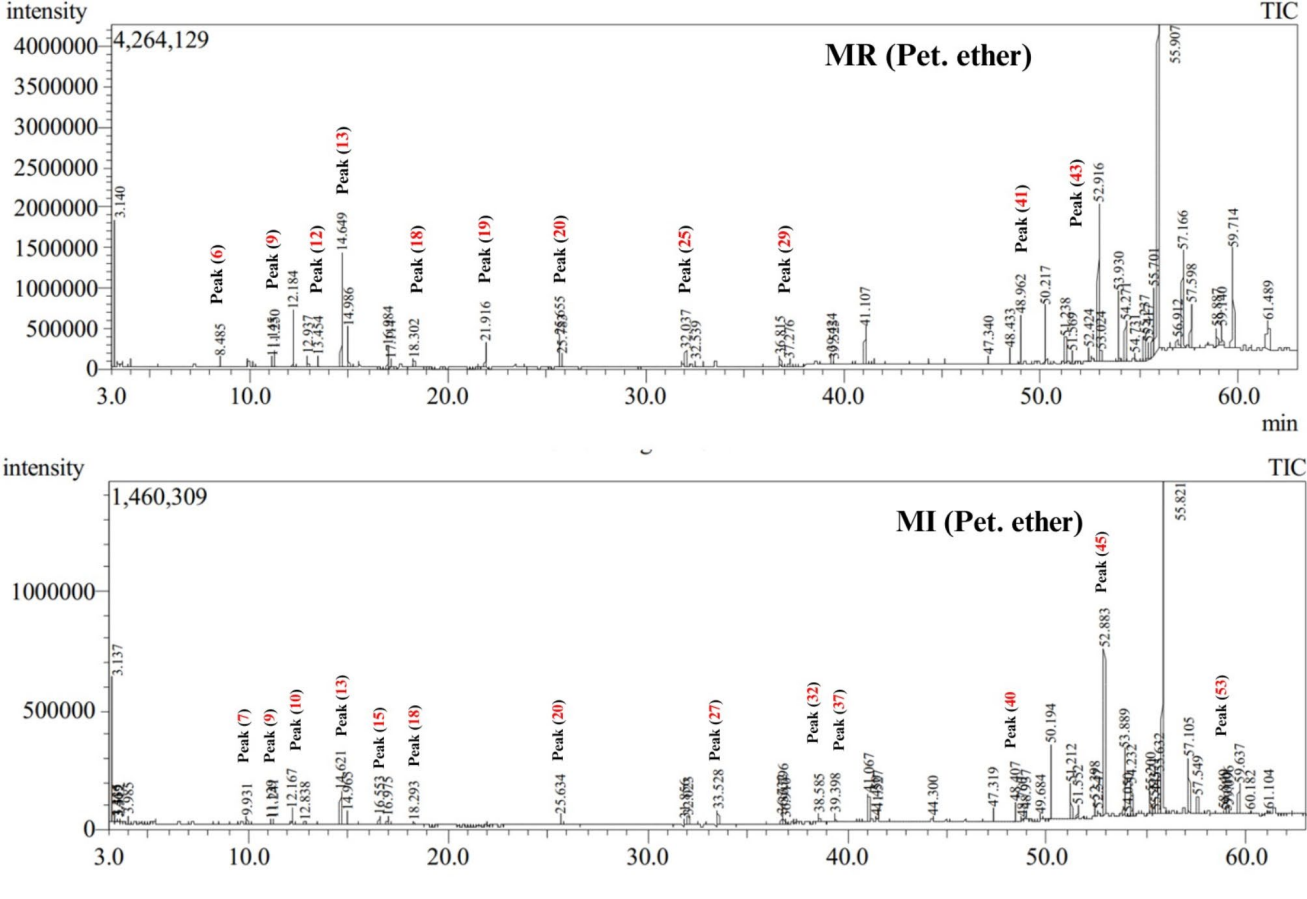
Representative GC chromatogram of volatiles identified in marjoram commercial products (MR and MI) analyzed after extraction with petroleum ether and peak numbers as listed in Table 2, peak (6): *β*-Phellandrene, peak (7): *p*-Cymene, peak (9): *γ*-Terpineol, peak (10): 2,4-Dimethylundecane, peak (12): 4-Thujanol, peak (13): Terpinen-4-ol, peak (15): Carvenone, peak (18): 4-Terpinenyl acetate, peak (19): Caryophyllene, peak (20): 10,12-Tricosadiynoic acid, methyl ester, peak (25): Neophytadiene, peak (27): Palmitic acid, methyl ester, peak (29): Methyl linolenate, peak (32): Palmitic acid, butyl ester, peak (37): n-Propyl linolenate, peak (40): Tetratriacontane, peak (41): *trans*-Geranylgeraniol, peak (43): Octacosane, peak (45): Pentatriacontane, peak (52): Tetracontane, peak (53): Tetratetracontane.

**Fig. 3:**
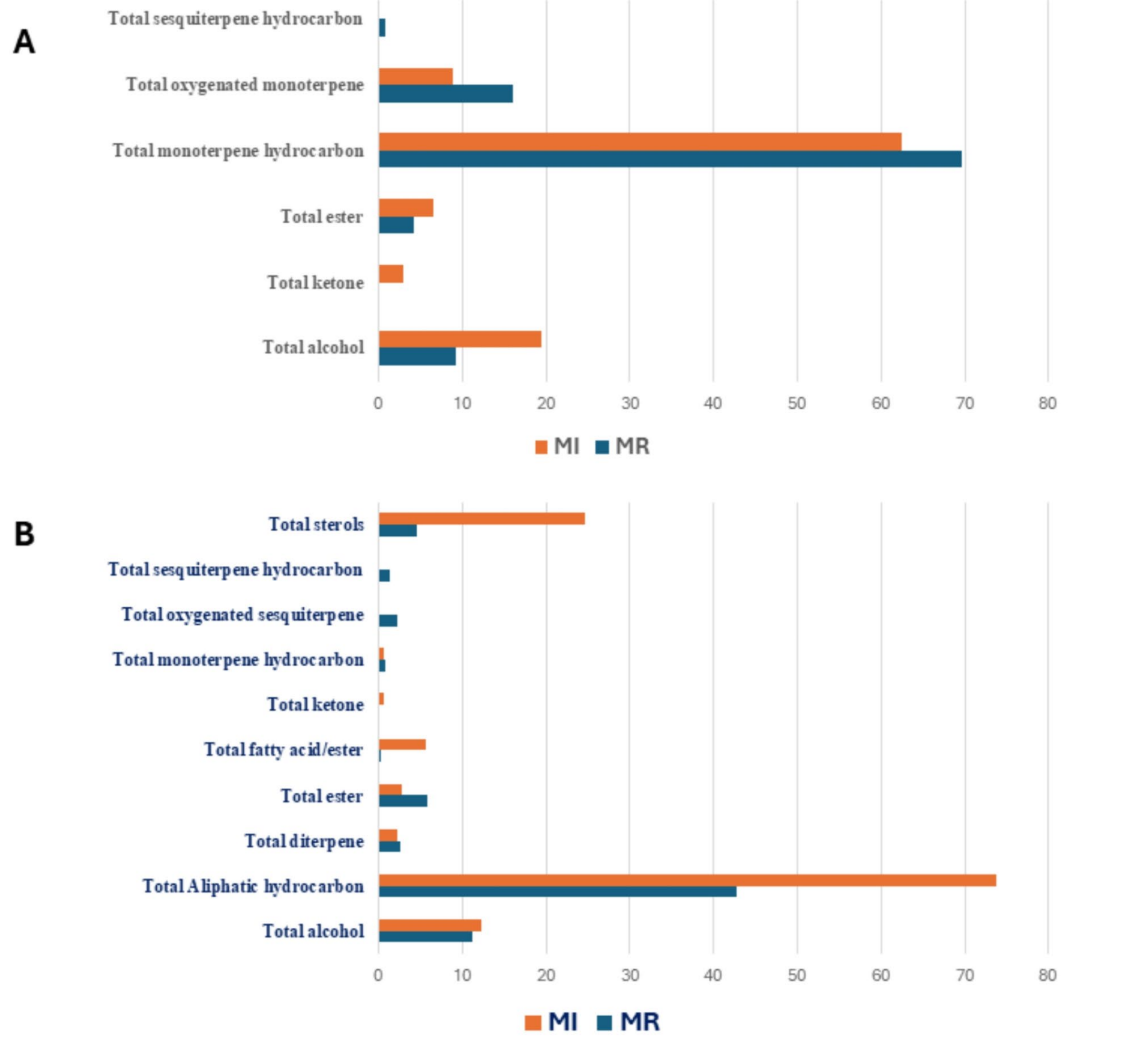
different volatile classes detected in marjoram samples (MR and MI) (**A**) marjoram samples analyzed by HS-SPME GC-MS. (**B**) Marjoram petroleum ether extract analyzed by GC-MS.

Analysis of the volatile profile of marjoram samples from two commercial sources extracted with petroleum ether using GC-MS analysis revealed the identification of 51 metabolites (Fig. 2; Table 2). The identified volatiles belonged to aliphatic hydrocarbons, alcohols, esters, monoterpene and sesquiterpene hydrocarbons, oxygenated sesquiterpenes, fatty acid/esters, ketones, and sterols (Fig. 3).

**Table 2:** Relative area percentage (%) of volatile metabolites in two commercial marjoram leaf products petroleum ether extract analyzed via head space GC-MS (n = 3).

Peak no	Rt	RI	Compound name	Class	MR (%)	sd	MI (%)	sd
1	3.137	794	3-Hexen-1-ol	Alcohol	3.25	0.02	5.14	0.00
9	11.25	1191	*γ*-Terpineol	Alcohol	2.69	0.03	0.28	0.01
12	12.937	1041	4-Thujanol	Alcohol	0.89	0.02	0.00	0.00
13	14.649	1137	Terpinen-4-ol	Alcohol	4.37	0.02	2.08	0.01
28	36.732	2052	Linoleyl alcohol	Alcohol	0.00	0.00	0.58	0.01
50	55.632	3942	1-Heptatriacotanol	Alcohol	0.00	0.00	4.12	0.04
Total alcohol					11.20	0.09	12.20	0.07
3	3.469	752	3-Methylheptane	Aliphatic hydrocarbon	0.00	0.00	0.15	0.01
4	3.512	842	1,3-Dimethylcyclohexane	Aliphatic hydrocarbon	0.00	0.00	0.12	0.01
5	3.985	816	Octane	Aliphatic hydrocarbon	0.00	0.00	0.35	0.02
10	12.838	1185	2,4-Dimethylundecane	Aliphatic hydrocarbon	0.00	0.00	0.17	0.02
23	31.856	2027	3-Eicosyne	Aliphatic hydrocarbon	0.00	0.00	0.33	0.02
31	37.276	3508	17-Pentatriacontene	Aliphatic hydrocarbon	0.27	0.01	0.00	0.00
39	47.34	2009	Eicosane	Aliphatic hydrocarbon	0.87	0.02	1.65	0.01
40	48.407	3401	Tetratriacontane	Aliphatic hydrocarbon	6.07	0.14	10.91	0.05
42	49.684	1448	5-Methyltetradecane	Aliphatic hydrocarbon	0.00	0.00	0.22	0.02
43	52.4	2080	Octacosane	Aliphatic hydrocarbon	0.58	0.02	2.33	0.03
45	52.883	3500	Pentatriacontane	Aliphatic hydrocarbon	8.78	0.01	11.11	0.02
46	53.889	3600	Hexatriacontane	Aliphatic hydrocarbon	9.62	0.05	10.01	0.03
49	55.417	1746	3-Methylheptadecane	Aliphatic hydrocarbon	0.91	0.01	0.05	0.03
52	55.821	3997	Tetracontane	Aliphatic hydrocarbon	12.83	0.02	35.02	0.09
53	58.849	4395	Tetratetracontane	Aliphatic hydrocarbon	2.85	0.02	1.36	0.03
Total aliphatic hydrocarbon					42.79	0.31	73.77	0.38
25	32.037	1774	Neophytadiene	Diterpene	0.72	0.01	0.00	0.00
35	41.067	2247	Dehydroabietinol	Diterpene	1.78	0.01	2.15	0.02
Total diterpene					2.50	0.02	2.15	0.02
14	14.986	1333	*α*-Terpinyl acetate	Ester	1.44	0.03	0.76	0.01
16	16.975	1381	*γ-*Terpinyl acetate	Ester	0.82	0.02	0.47	0.02
17	17.114		Linalyl acetate	Ester	0.32	0.02	0.00	0.00
18	18.3	1327	4-Terpinenyl acetate	Ester	0.26	0.01	0.17	0.02
33	39.425	2370	4-epi-Dehydroabietinol acetate	Ester	0.55	0.02	0.00	0.00
34	39.525	2404	Butyl citrate	Ester	0.21	0.01	0.00	0.00
36	41.45	2193	Linoleyl acetate	Ester	0.00	0.00	0.18	0.02
41	48.937	2192	*trans*-Geranylgeraniol	Ester	2.15	0.02	0.61	0.05
24	32.023	4085	1,1-Bis(dodecyloxy)hexadecane	Ether	0.00	0.00	0.53	0.02
Total ester					5.74	0.12	2.72	0.13
20	25.634	2609	10,12-Tricosadiynoic acid, methyl ester	Fatty acid/ester	0.00	0.00	0.73	0.02
27	33.528	1878	Palmitic acid, methyl ester	Fatty acid/ester	0.00	0.00	0.95	0.02
29	36.815	2101	Methyl linolenate	Fatty acid/ester	0.34	0.01	1.27	0.02
30	36.91	1886	7-Hexadecenoic acid, methyl ester	Fatty acid/ester	0.00	0.00	0.38	0.01
32	38.585	2177	Palmitic acid, butyl ester	Fatty acid/ester	0.00	0.00	0.57	0.02
37	41.527	2300	n-Propyl linolenate	Fatty acid/ester	0.00	0.00	0.56	0.02
48	55.371	2980	Eicosyl nonyl ether	Fatty acid/ester	0.00	0.00	1.48	0.03
Total fatty acid/ester					0.34	0.01	5.95	0.14
15	16.553	1158	Carvenone	Ketone	0.00	0.00	0.54	0.02
Total ketone					0.00	0.00	0.54	0.02
6	8.485	964	*β*-Phellandrene	Monoterpene hydrocarbon	0.38	0.01	0.00	0.00
7	9.931	1042	*p*-Cymene	Monoterpene hydrocarbon	0.00	0.00	0.45	0.03
8	11.145	998	*γ*-Terpinene	Monoterpene hydrocarbon	0.36	0.03	0.22	0.01
Total monoterpene hydrocarbon					0.74	0.04	0.67	0.04
21	25.655	1569	Isospathulenol	Oxygenated sesquiterpene	1.33	0.02	0.00	0.00
22	25.783	1507	Caryophyllene oxide	Oxygenated sesquiterpene	0.56	0.02	0.00	0.00
47	54.731	1484	Cubebol	Oxygenated sesquiterpene	0.34	0.02	0.00	0.00
Total oxygenated sesquiterpene					2.23	0.06	0.00	0.00
19	21.916	1494	β-Caryophyllene	Sesquiterpene hydrocarbon	1.03	0.02	0.00	0.00
26	32.539	1393	Longipinane	Sesquiterpene hydrocarbon	0.28	0.01	0.00	0.00
Total sesquiterpene hydrocarbon					1.31	0.03	0.00	0.00
51	55.701	2731	*γ*-Sitosterol	Sterol	4.55	0.00	24.63	0.25
Total sterol					4.55	0.00	24.63	0.25

Significance value bold.

#### Aliphatic hydrocarbons

Aliphatic hydrocarbons represented by 15 peaks were detected as the major metabolite class detected in marjoram samples extracted with petroleum ether to account for ca. 73.8 and 42.8% in MI and MR samples, respectively. Tetracontane (peak 52) was the major aliphatic hydrocarbon detected in marjoram samples at a higher level in MI sample at 35.02% compared to 12.8% in MR samples. Moreover, other contane-derivatives including tetratriacontane (peak 40), pentatriacontane (peak 45), and hexatriacontane (peak 46) were detected at a level range 6.1–9.6% in MR compared to a higher-level range at 10.01–11.1% in MI samples.

#### Alcohol, ketone, and esters

Alcohols were detected at comparable levels of 11–12% in marjoram samples MR and MI. Compared to SPME extraction, several alcohols were detected including 3 -hexen-1-ol, 1-heptatriacontanol, linalyl alcohol, terpene-4-ol, 4-thujanol, and *γ*-terpineol. 3-Hexen-1-ol (peak 1) was detected at a high level of 5.1% in MI sample compared to 3.3% in MR sample, whereas terpene-4-ol (peak 13) was detected in MR at a higher level (4.4%) than MI samples (2.1%). Fatty alcohols including 1-heptatriacontanol (peak 50) and linoleyl alcohol (peak 28) were detected only in MI samples extracted with petroleum ether. In accordance with SPME results, carvenone was detected as a major ketone in marjoram samples MI. Esters represented by 9 peaks were detected at 5.7% in MR compared to 2.7% in MI. Geranylgeraniol (peak 41) and terpinyl acetate (peak 14) were the abundant esters.

#### Monoterpene, diterpenes, and sesquiterpene hydrocarbons

Monoterpene hydrocarbons represented by *β*-phellandrene, p-cymene, and *γ*-terpinene were detected at trace levels in both marjoram samples extracted with petroleum ether. Likewise, diterpenes including neophytadiene and dehydroabietinol were detected at 2.1–2.5% in marjoram samples. Sesquiterpene hydrocarbons were detected only in MR samples (1.3%), and in accordance with the SPME extraction method. Caryophyllene (peak 19) and longipinane (peak 26) represented the main sesquiterpenes detected in MR extracted with petroleum ether, versus santalene detected using SPME extraction.

#### Fatty acids/esters and sterols

Fatty acids were detected at high levels in marjoram samples MI 5.6% compared with trace levels in MR samples indicating the effect of solvent towards recovery of nonpolar compounds in marjoram. Sterols were detected at higher levels in MI samples at 24.6% represented by *γ*-Sitosterol compared to a much lower level of 4.5% in MR samples.

### PCA analysis of marjoram volatile metabolites analyzed with GC-MS

**Fig. 4:**
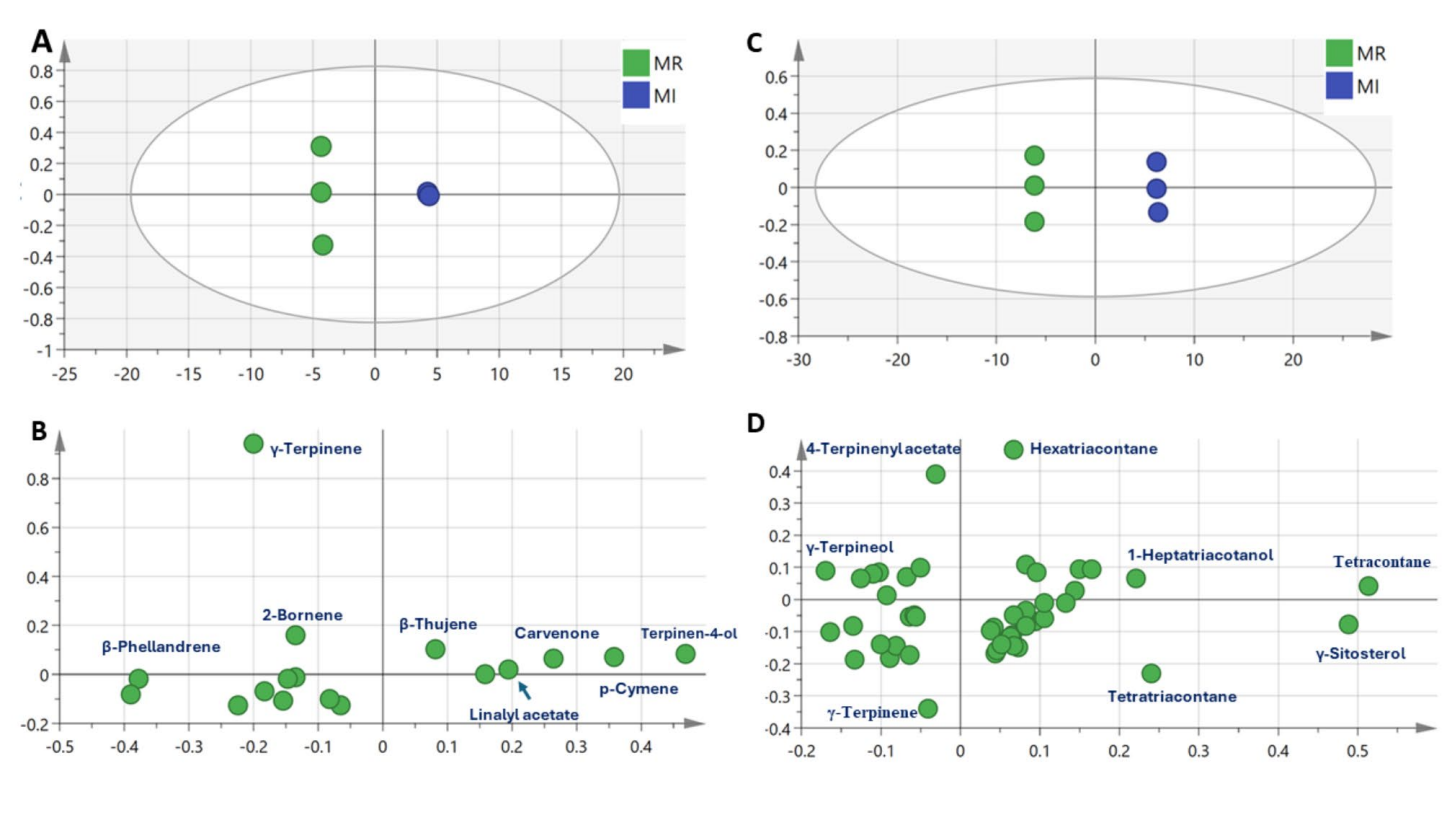
Unsupervised multivariate data analyses of two marjoram samples volatile compounds detected using GC-MS (*n* = 3). (**A**) PCA score plot of PC1 vs. PC2 scores of samples analyzed by HS-SPME GC-MS. (**B**) The respective loading plot for PC1 and PC2, provide peak assignments. (**C**) PCA score plot of PC1 vs. PC2 scores of samples analyzed by petroleum ether extraction coupled with GC-MS. (**D**) The respective loading plot for PC1 and PC2, provide peak assignments. The metabolite clusters are placed in two-dimensional space at the distinct locations defined by two vectors of principal component.

Multivariate data analysis using principal component analysis (PCA) was further used for better assessment of volatiles distribution among marjoram samples from two commercial sources (Fig. 4). A PCA model (Fig. 4A) of marjoram volatiles extracted by HS-SPME showed discrimination of MR clusters at the left side of PC1 versus MI samples clustering towards the right side of PC1. The corresponding loading plot (Fig. 4B) revealed that *γ*-terpinene, *β*-phellandrene, and 2-bornene were more enriched in MR samples, versus abundance of oxygenated terpenes i.e., terpinen-4-ol, carvenone, and linalyl acetate in MI samples, in addition to *β*-thujene and cymene and accounting for its segregation. The abundance of monoterpene hydrocarbon and oxygenated compound in samples extracted using HS-SPME revealed the efficiency of the extraction technique for evaluation of the quality of sweet oregano as it is in agreement with the hydro-distillation method^7^.

Another PCA model (Fig. 4C) of marjoram volatiles extracted by petroleum ether showed discrimination of MR clusters at the left side of PC1 while MI samples were clustered towards the right side of PC1. The corresponding loading plot Fig. 4D revealed that *γ*-terpinene, 4-terpinenyl acetate, and *γ*-terpineol were enriched in MR samples. Aliphatic hydrocarbons including tetracontane, hexatriacontane, tetratriacontane along with 1-heptatriacotanol and *γ*-sitosterol were more enriched in MI samples, and to account for their segregation toward the right side. However, the extraction of marjoram using petroleum ether method yielded a significant number of metabolites, including many non-polar compounds, these non-polar compounds could potentially interfere with the quality assessment of marjoram samples. Hence, HS-SPME appears as the most suitable for assessment of marjoram samples quality.

### Total phenolics and flavonoids in marjoram from two commercial sources

The total phenolics content assay in the two marjoram samples revealed comparable levels of 109–112 µg GA/mg in MR samples (Table 3). Likewise, total flavonoid content was detected at 18.3 µg rutin eq/mg in MR samples compared with 19.5 µg rutin eq/mg in MI samples. Compared to a previous report on Spanish marjoram total phenolics revealed that marjoram contained 163 mg GAE/L^34^. Profiling of Egyptian marjoram showed the presence of apigenin, methyl rosmarenate, rosmarenic acid, luteolin-7-*O*-rutinose, as a major compound, meanwhile *p*-coumaric acid, gallic acid, chlorogenic acid, caffeic acid, and ferulic acid were detected using HPLC analysis^35^. Additionally, Tunisian marjoram aerial parts revealed the presence of phenolic acids viz. (E)-2-hydroxycinnamic, rosmarinic, vanillic, chlorogenic, gallic, and cinnamic, whereas flavonoids including amentoflavone, apigenin, quercetin, luteolin, coumarin, and rutin^36^.

**Table 3:** Results of total phenolic contents, total flavonoid content, and DPPH radical scavenging capacity of two commercial Marjoram products (R and I) methanolic extract.

Sample	Total phenolic content (µg GA/mg sample)	Total flavonoid content (µg rutin eq/mg sample)	DPPH IC_50_ (µg/mL)
MR	111.88 ± 3.11	18.32 ± 0.98	45.53 ± 0.66
MI	109.11 ± 8.99	19.54 ± 1.49	56.78 ± 0.86
Trolox (µg/mL)			6.57 ± 0.45

### DPPH radical scavenging capacity of marjoram methanol extracts

**Fig. 5:**
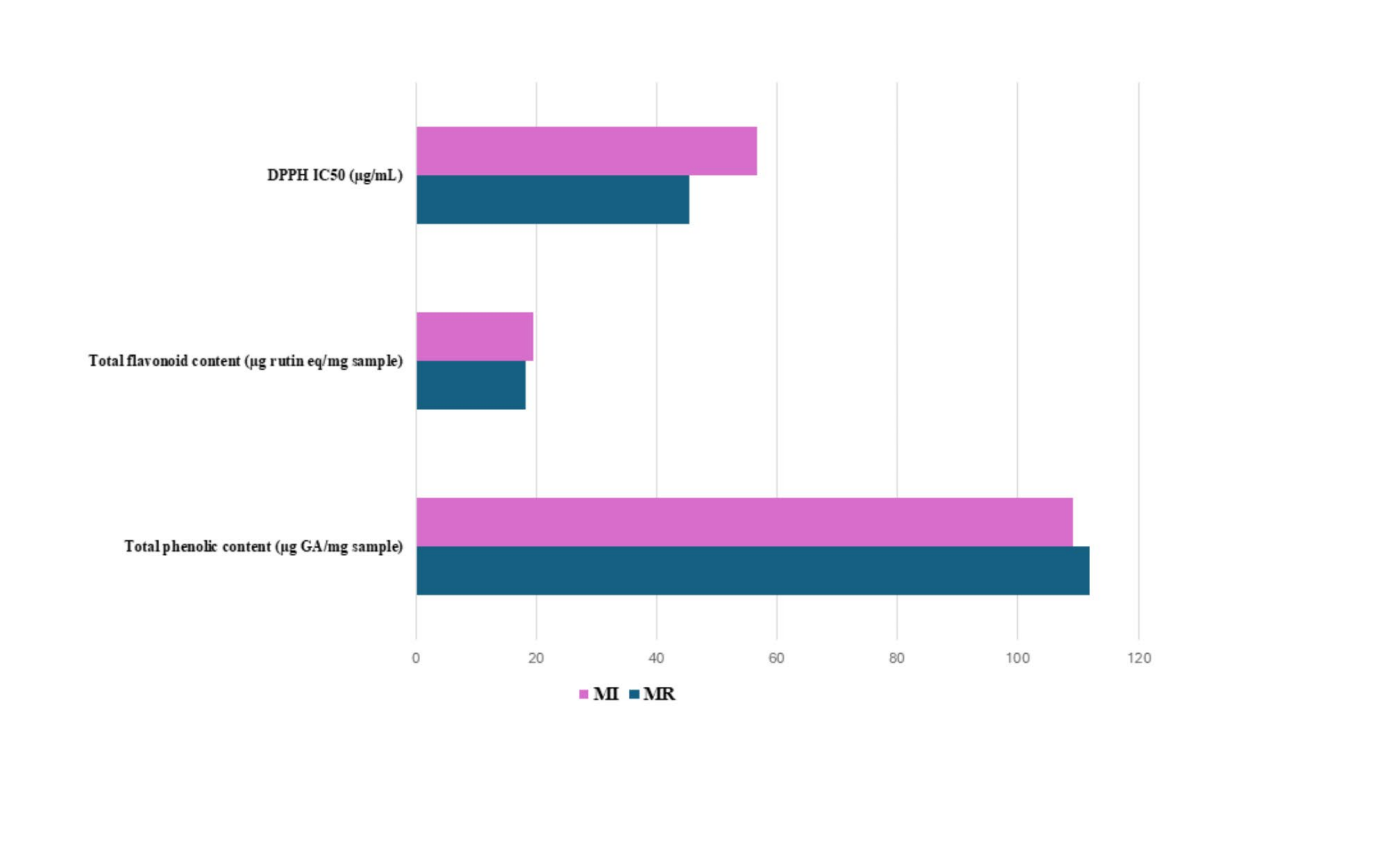
Total phenolic and flavonoid content and % inhibition of DPPH radical scavenging capacity of methanol extract of marjoram samples from two commercial sources (MR and MI).

Investigation of DPPH radical scavenging capacity revealed that MR samples showed radical scavenging capacity with an IC_50_ value of 45.5 µg/mL compared with 56.8 µg/mL for MI samples indicating the slightly stronger antioxidant effect of MR (Table 3; Fig. 5), likely attributed to its richness in phenolics and terpenes^34^. Next to phenolics, predominant monoterpene hydrocarbons viz. *β*-phellandrene, *p*-cymene, and *γ*-terpinene detected in marjoram essential oil may also contribute to antioxidant activity^34^. In agreement with other previous studies, Tunisian *O. majorana* revealed high antioxidant activity^37^. Moreover, Iranian *O. majorana* revealed a similar composition of *cis*-sabinene hydrate and terpineol, with potential antioxidant capacity^38^.

### Anti-bacterial activities of petroleum ether extract of marjoram samples from two commercial products

Essential oil-containing herbal extracts are well recognized for their potential antibacterial activity against both Gram-positive and Gram-negative bacteria and the results compared to gentamycin as a standard antibacterial drug^39^. Consequently, the antibacterial activity of marjoram samples was tested against 5 Gram-positive bacterial strains and 3 Gram-negative strains (Table 4). Both marjoram samples revealed moderate antibacterial activities as concluded from the zone of inhibition compared to standard antibacterial agents. MR and MI extracts showed activity against *Bacillus subtilis* with an inhibition zone of 16.03 and 15.9 mm, respectively. Against *Bacillus cereus*, MR extract showed an inhibition zone of 12.9 mm compared with 13.7 mm for MI extract. Moreover, both extracts showed an inhibition effect against *Enterococcus faecalis* with a comparable inhibition zone of 14.03 mm. Both *Staphylococcus aureus* and *Streptococcus mutants* were found resistant against both marjoram extracts. Regarding Gram-negative bacteria, both marjoram extracts showed an inhibition only against *Enterobacter cloacae* with an inhibition zone of 11.6 mm. The antibacterial activity of marjoram oil was previously investigated against *S. aureus*, *E. faecalis*, *E. coli*, and *K. pneumoniae*^40^. Results revealed that among volatiles in *O. majorana* essential oil, *cis*-sabinene hydrate contributed towards the inhibition of bacterial growth^40^. In another study, Pakistani sweet marjoram exhibited antibacterial activity against *S. aureus*, *B. cereus*, *B. subtilis*, *P. aeruginosa*, and *E. coli*^31^.

**Table 4:** Comparative antibacterial activity of two marjoram commercial products (MR and MI) petroleum ether extract expressed as zone of inhibition (mm).

Evaluated microorganism/sample	MR (Av ± sd)	MI (Av ± sd)	Gentamycin
Gram-positive bacteria			
* Staphylococcus aureus*	N/A	N/A	24.01 ± 0.01
* Bacillus subtilis*	16.03 ± 0.35	15.91 ± 0.40	26.02 ± 0.03
* Streptococcus mutants*	N/A	N/A	20.01 ± 0.02
* Bacillus cereus*	12.97 ± 0.25	13.71 ± 0.40	24.97 ± 0.06
* Enterococcus faecalis*	14.03 ± 0.45	13.97 ± 0.35	26.09 ± 0.01
Gram-negative bacteria			
* Enterobacter cloacae*	11.60 ± 0.53	11.56 ± 0.50	27.03 ± 0.06
* Escherichia coli*	N/A	N/A	29.90 ± 0.01
* Salmonella typhimurium*	N/A	N/A	17.02 ± 0.03

## Material and methods

### Sample collection

Two different commercial products packaged with dried sweet marjoram (*Origanum majorana* L.) leaf produced by two different companies namely Royal (MR (and Isis (MI) were collected from a local market in Cairo, Egypt.

### Sample preparation

#### HS-SPME

Fibers used in SPME volatile extraction are stable flex coated with divinylbenzene/carboxen/polydimethylsiloxane (DVB/CAR/PDMS, 50/30 µm) or PDMS (polydimethylsiloxane) and were purchased from Supelco (Oakville, ON, Canada). Volatile standards were provided by Sigma Aldrich (St. Louis, MO, USA). Marjoram samples (100 mg) were placed in an SPME screw-cap vial (1.5mL) spiked with 10 µg (*Z*)-3-hexenyl acetate with fibers inserted manually above and placed in an oven kept at 50 °C for 30 min. HS-SPME analysis of the volatile compounds was performed as reported in^41^ with slight modifications. The fiber was subsequently withdrawn into the needle and then injected manually into the injection port of a gas chromatography–mass spectrometer (GC–MS).

#### Solvent extraction using petroleum ether

The powdered marjoram leaf sample (20 g each) was extracted with petroleum ether (150 mL × 3) with cold sonication at 35 °C (using a Biomall sonicator, India) for volatiles extraction. Petroleum ether was selected considering its non-polar nature more suited to recovering volatile terpenes with less interference from other less volatile phytochemicals^39^. The petroleum ether extract was filtered and concentrated under reduced pressure using a Rotary evaporator (Hahin-shin, Japan) at 40 °C till the complete evaporation of petroleum ether to yield concentrated extract used for GC-MS analysis.

### GC-MS analysis of marjoram aroma profile

GC/MS analysis was performed using Shimadzu GCMS-QP2020 (Tokyo, Japan). The GC was equipped with Rtx-1MS fused bonded column (30 m × 0.25 mm i.d. × 0.25 μm film thickness) (Restek, USA) and a split–splitless injector. The initial column temperature was kept at 45 °C for 2 min (isothermal) and programmed to 300 °C at a rate of 5 °C/min and kept constant at 300 °C for 5 min (isothermal). The injector temperature was 250 °C. The helium carrier gas flow rate was 1.41 ml/min. All the mass spectra were recorded applying the following conditions: (equipment current) filament emission current, 60 mA; ionization voltage, 70 eV; ion source, 200 °C. Diluted samples (1% v/v) were injected with split mode (split ratio 1: 15).

### Identification of volatile chemical composition

Identification was based on the calculation of retention indices of each component, mass spectra matching with NIST-11 and Wiley library database as well as published data in literature^31,42^. Retention indices (RI) were calculated relative to a homologous series of n-alkanes (C8-C28) injected under the same conditions.

### Total phenolic content assay

The total phenolic content was determined using the Folin–Ciocalteu method^43^. Samples were prepared at the concentration of 4 mg/mL in methanol. Briefly, the procedure consisted of mixing 10 µL of sample/standard with 100 µL of Folin-Ciocalteu reagent (Diluted 1: 10) in a 96-well microplate. Then, 80 µL of 1 M Na_2_CO_3_ was added and incubated at room temperature (25 °C) for 20 min in the dark. At the end of incubation time, the resulting blue complex color was measured at 630 nm. Data are represented as means ± SD. A Gallic acid standard curve was used for the calculation of total phenolic content. The results were recorded using a microplate reader FluoStar Omega.

### Total flavonoid content assay

The total flavonoids level was determined using the aluminum chloride method^44^, with minor modifications. Samples were prepared at 4 mg/mL in methanol. Briefly, 15µL of sample/standard was placed in a 96-well microplate, then, 175 µL of methanol was added followed by 30 µL of 1.25% AlCl_3_. Finally, 30 µL of 0.125 M C_2_H_3_NaO_2_ was added and incubated for 5 min. At the end of the incubation time, resulting yellow color was measured at 420 nm. Data are represented as means ± SD. Rutin standard curve was used for the calculation of total flavonoid content. The results were recorded using a microplate reader FluoStar Omega.

### DPPH free radical scavenging capacity assay

DPPH (2,2-diphenyl-1-picryl-hydrazyl-hydrate) free radical assay was carried out according to the method of^45^. Briefly, 100µL of freshly prepared DPPH reagent (0.1% in methanol) was added to 100µL of the sample in a 96-well plate (*n* = 6), and the reaction was incubated at room temp for 30 min in the dark. At the end of incubation time, the resulting reduction in DPPH color intensity was measured at 540 nm. Data are represented as means ± SD according to the following Eq. (1): percentage inhibition= ((Average absorbance of blank-average absorbance of the test)/(Average absorbance of blank))*100.

Trolox standard curve was used as a positive control for antioxidant assay. The results were recorded using a microplate reader FluoStar Omega. Samples MI, and MR were prepared at final concentrations of 15.625, 31.25, 62.5, 125, and 250 µg/ml in Methanol. Trolox stock solution of 20 µg/mL was prepared in 100% methanol from which 5 concentrations were prepared including 10, 7.5, 5, 2.5, and 1.25 µg/mL.

### In vitro antibacterial activity

The antibacterial activity of tested samples was investigated in vitro against eight bacterial strains and results were expressed as zone of inhibition) mm). The culture medium was Mueller-Hinton agar recommended by the National Committee for Clinical Laboratory Standards. The standard antimicrobial agent was gentamicin; an antibacterial agent obtained from the Regional Center for Mycology and Biotechnology, Al-Azhar University, Egypt. The microorganisms were obtained from the Regional Center for Mycology and Biotechnology, Al-Azhar University, Egypt. Gram-positive bacteria *Staphylococcus aureus* ATCC 25923, *Bacillus subtilis* RCMB 015 (1) NRRL B-543, *Bacillus cereus* RCMB 027 (1), *Streptococcus mutants RCMB* 017 (1) ATCC 25175, *Enterococcus faecalis* ATCC 29212. *Gram-negative bacteria Enterobacter cloacae* RCMB 001(1) ATCC 23355, *Escherichia coli* ATCC 25922, *and Salmonella typhimurium* RCMB 006 (1) ATCC 14028.

### Statistical analysis

The results were displayed as mean ± standard deviation of the mean (SD). Data was analyzed using Microsoft Excel and the IC_50_ value was calculated using Graph pad Prism 6.07 (https://www.graphpad.com) by converting the concentrations to their logarithmic value and selecting non-linear inhibitor regression Eq. (2): (log (inhibitor) vs. normalized response – variable slope equation).

## Conclusion

The current work aims to assess volatile metabolite variations in marjoram samples collected from two commercial products in Egypt for the first time using two different extraction methods viz. HS-SPME and petroleum ether and analysed using GC-MS analysis. A total of 20 and 51 volatile metabolites were identified in samples extracted with HS-SPME and petroleum ether, respectively. HS-SPME revealed the abundance of monoterpene hydrocarbons and oxygenated compounds. The major identified aroma compounds were *β*-phellandrene (20.1 and 14.2%), *γ*-terpinene (13.4 and 11.7%), 2-bornene (12.3 and 11.5%), *p*-cymene (9.8% and 4.6%) terpenen-4-ol (16.4% and 7.5%), sabinene hydrate (16.02% and 8.8%) and terpineol (4.2 and 3.2%) in MR and MI, respectively. Extraction with petroleum ether revealed the abundance of aliphatic hydrocarbons (42.8 and 73.8%) in MR and MI, respectively. PCA analysis revealed variation in volatiles between both samples with a distinct quality in MR samples with higher *cis*-sabinene hydrate. The abundance of monoterpenes and oxygenated compounds in HS-SPME samples revealed the efficiency of the extraction in marjoram essential oil composition assessment, while petroleum ether increased the recovery of non-polar compounds. The total phenolics and flavonoids in marjoram samples were (111.9, 109.1 µg GA/mg) and (18.3, 19.5 µg rutin eq/mg) in MR and MI, respectively. MR samples showed DPPH inhibition with IC_50_ at 45.5 µg/mL compared with 56.8 µg/mL for MI samples inferring for an antioxidant effect. The antibacterial activity of marjoram samples was tested against Gram-positive and Gram-negative strains, with moderate antibacterial activities. HS-SPME appears generally more suited for the quality assessment of marjoram essential oil. Further LC-MS-based analysis is recommended for profiling of phenolics and flavonoids in marjoram to likely account for its antioxidant effects.

## Data Availability

All data generated or analyzed during this study are included in this published article.
